# Single institution experience of MRI-guided radiotherapy for thoracic tumors and clinical characteristics impacting treatment duty cycle

**DOI:** 10.3389/fonc.2024.1401703

**Published:** 2024-06-11

**Authors:** Joseph A. Miccio, Nicholas J. Potter, Anaum Showkat, Min Yao, Sean Mahase, Michele Ferenci, Kaitlin Sisley, Amy Dailey, Jamie Knipple, Amy Blakely, Leonard Tuanquin, Mitchell Machtay

**Affiliations:** ^1^Department of Radiation Oncology, Penn State Cancer Institute, Hershey, PA, United States; ^2^Department of Arts and Letters, University of Notre Dame, South Bend, IN, United States

**Keywords:** MRI guidance, radiotherapy, thoracic, SBRT, duty cycle

## Abstract

**Introduction:**

MRI-guided radiotherapy (MRgRT) allows for direct motion management and real-time radiation treatment plan adaptation. We report our institutional experience using low strength 0.35T MRgRT for thoracic malignancies, and evaluate changes in treatment duty cycle between first and final MRgRT fractions.

**Methods:**

All patients with intrathoracic tumors treated with MRgRT were included. The primary reason for MRgRT (adjacent organ at risk [OAR] vs. motion management [MM] vs. other) was recorded. Tumor location was classified as central (within 2cm of tracheobronchial tree) vs. non-central, and further classified by the Expanded HILUS grouping. Gross tumor volume (GTV) motion, planning target volume expansions, dose/fractionation, treatment plan time, and total delivery time were extracted from the treatment planning system. Treatment plan time was defined as the time for beam delivery, including multileaf collimator (MLC) motion, and gantry rotation. Treatment delivery time was defined as the time from beam on to completion of treatment, including treatment plan time and patient respiratory breath holds. Duty cycle was calculated as treatment plan time/treatment delivery time. Duty cycles were compared between first and final fraction using a two-sample t-test.

**Results:**

Twenty-seven patients with thoracic tumors (16 non-small cell lung cancer and 11 thoracic metastases) were treated with MRgRT between 12/2021 and 06/2023. Fifteen patients received MRgRT due to OAR and 11 patients received MRgRT for motion management. 11 patients had central tumors and all were treated with MRgRT due to OAR risk. The median dose/fractionation was 50 Gy/5 fractions. For patients treated due to OAR (n=15), 80% had at least 1 adapted fraction during their course of radiotherapy. There was no plan adaptation for patients treated due to motion management (n=11). Mean GTV motion was significantly higher for patients treated due to motion management compared to OAR (16.1mm vs. 6.5mm, *p*=0.011). Mean duty cycle for fraction 1 was 54.2% compared to 62.1% with final fraction (*p*=0.004). Mean fraction 1 duty cycle was higher for patients treated due to OAR compared to patients treated for MM (61% vs. 45.0%, *p*=0.012).

**Discussion:**

Duty cycle improved from first fraction to final fraction possibly due to patient familiarity with treatment. Duty cycle was improved for patients treated due to OAR risk, likely due to more central location and thus decreased target motion.

## 1 Introduction

Lung cancer is the third most common malignancy in the United States (1), and the lung is one of the most common sites of metastatic spread for from other primary cancers (2). The safe and effective delivery of radiotherapy to intrathoracic tumors is challenging due to critical organs at risk (OARs) such as the bronchial tree, esophagus, heart, and spinal cord as well as inherent respiratory motion leading to uncertainty in tumor position throughout the respiratory cycle (3). Even modern series evaluating radiotherapy for tumors near the bronchial tree have shown unacceptable grade 5 toxicity rates of up to 15% (4).

Magnetic Resonance Image Guided Radiotherapy (MRgRT) utilizing a 0.35T MR-Linac allows for direct visualization of targets during treatment and motion-management by way of patient breath hold gating (i.e. delivery of radiation at a particular position in the patient’s respiratory cycle). Additionally, the system allows for online real-time dosimetric evaluation, and the ability to adapt the radiation plan based on daily anatomic changes to optimize target coverage while ensuring acceptable dose to OARs. MRgRT offers a unique solution to safely and effectively treat intrathoracic tumors by accounting for adjacent OARs and respiratory motion (5).

This manuscript describes our experience treating thoracic tumors with 0.35T MRgRT. Herein we describe our experience including patient demographics and the clinical reasoning for utilizing MRgRT for each case. We also evaluate the frequency of radiation plan adaptation and finally we evaluate the change in treatment duty cycle between first and final fractions of radiotherapy. Lastly, we explore duty cycle differences between patients treated before and after our department installed an in-bore viewing video display to provide visual feedback to patients to assist with breath hold gating.

## 2 Methods

All patients with intrathoracic tumors treated with MRgRT from December 2021 through June 2023 were included. Institutional Review Board approval was obtained for this analysis (Study 00021052). All patients were treated with respiratory gating, and 19 patients were treated after the visual feedback display installation. The primary reason for MRgRT (OAR vs. motion management vs. other) was recorded by the treating physician. Tumor location was classified as central (within 2cm of tracheobronchial tree) vs. non-central, and further classified by the Expanded HILUS grouping (A: ≤ 1 cm from the mainstem bronchus; B: ≤ 1 cm from the lobar bronchi but >1 cm from the mainstem bronchi; C: 1 - 2 cm around the tracheobronchial tree; D: ≤ 1 cm from the trachea but > 2 cm from the carina.) (6). Gross tumor volume (GTV) motion during free breathing, planning target volume (PTV) expansions, and radiation dose and fractionation were recorded for each patient. For each fraction, OARs were evaluated online and contours were adjusted as needed. The radiation plan was predicted on the anatomy of the day, and the decision to adapt the plan was at the treating physician’s discretion. In general, plans were adapted to improve target coverage, decrease OAR dose, or both.

The treatment plan time (TPT) and total delivery time (TDT) were extracted from the treatment planning system for the first and final fractions of each patient’s treatment course. Treatment plan time is defined as the time for beam delivery, including multileaf collimator (MLC) motion and gantry rotation (i.e. time for treatment delivery if target was always within the treatment boundary). If there was no plan adaptation, the first fraction’s treatment plan time is equal to the final fraction’s treatment plan time. Treatment delivery time was defined as the time from beam-on to fraction completion (i.e. treatment plan time plus time for patient to repeatedly breath-hold the target into the treatment boundary).

The ratio of time spent by the signal within the specific portion of the respiratory cycle (“gate”) to the overall treatment time is referred to as the duty cycle (7). Beam gating for motion management on the 0.35T MR-Linear Accelerator is performed by having the patient hold their breath and tracking the physical location of the tumor on real-time cine MRI imaging. When the patient’s target is within the correct positional window the machine delivers radiation. Duty cycle is a measure of efficiency of this method. It is important to note the user can select two parameters during tracking that have a significant impact on duty cycle, size of positional window and %ROI-threshold (the acceptable ROI percentage outside of the tracking positional window). The positional window for all patients treated in this study was created by applying a 3 mm isotropic expansion on the tracking volume, and the %ROI-threshold was set between 5–8% for all patients, with majority utilizing 5%. A consistent definition of duty cycle for MRgRT is not well established (8–10). In the present study, Duty cycles (DC) were calculated for the first fraction (i), and final fraction (f) as:


$$DC_{i}=\frac{TPT_{i}}{TDT_{i}}$$

$$DC_{f}=\frac{TPT_{f}}{TDT_{f}}$$

Duty cycles were compared between first and final fraction, and between patients receiving MRgRT for primary reason of OAR vs. motion management using a two-sample t-test. The analysis was performed using STATA, version 13.1 (StataCorp LLC, College Station, TX) and P-values< 0.05 were considered statistically significant.

## 3 Results

**Figure 1:**
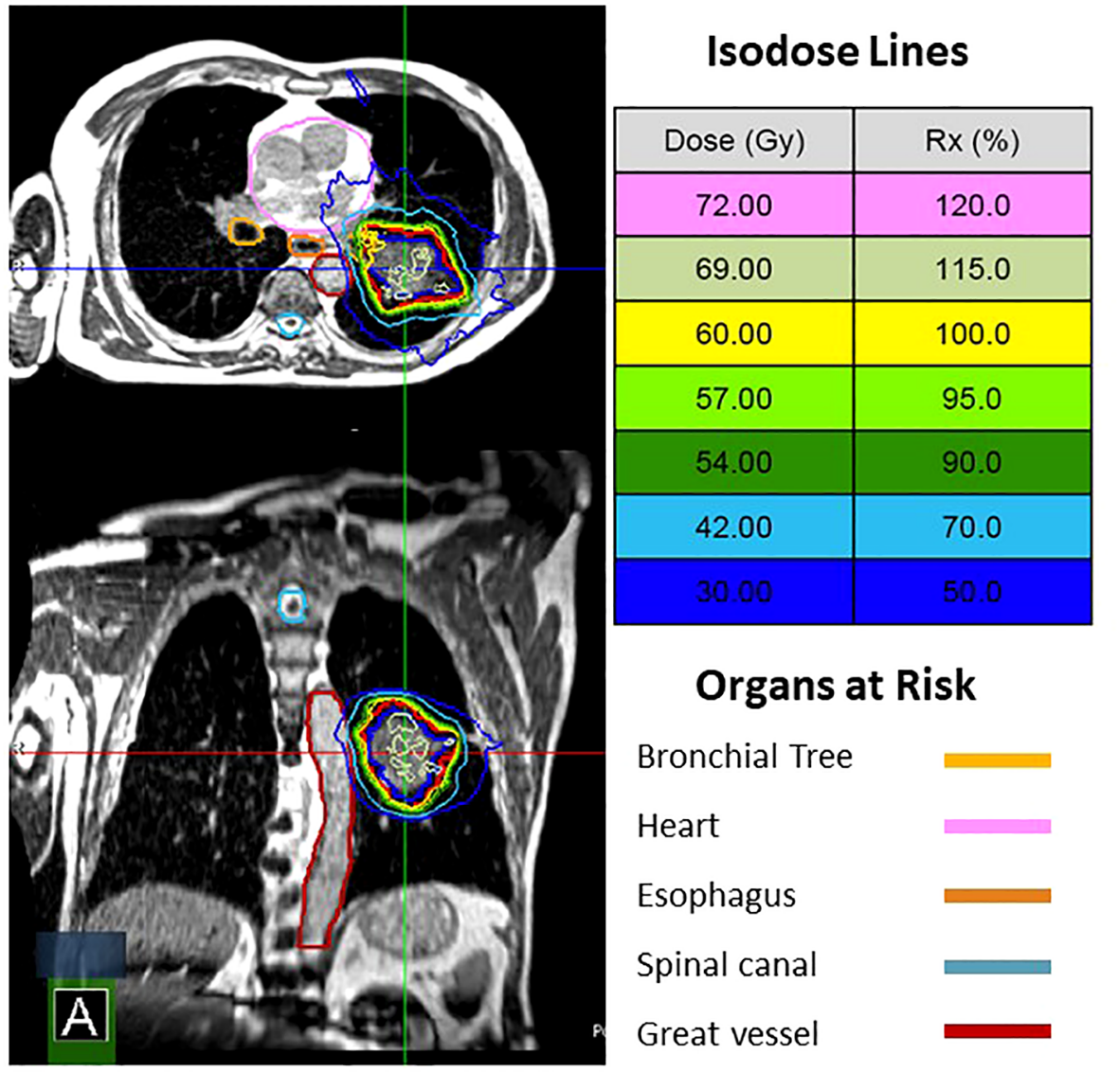
MRgRT plan for a patient with a cT3N0 NSCLC. He was not a surgical candidate. HILUS B due to proximity to the left upper lobe bronchus. The patient was treated with 60 Gy in 8 fractions with 1 of 8 fractions adapted.

Twenty-seven patients with thoracic disease were treated with a total of 169 fractions of MRgRT between December of 2021 and June of 2023 (**Table 1**). Patients treated with MRgRT had good to excellent performance status with 30% ECOG 0 and 70% ECOG 1. Sixteen patients had non-small cell lung cancer and 11 patients had metastatic disease to the chest from another primary site. The majority of treated lesions were within the lung parenchyma, however 4 patients were treated to mediastinal lymph nodes. Eleven patients met Expanded HILUS grouping definition (6 group A, 1 group B, 2 group C, and 2 group D). Fifteen patients were treated with MRgRT due to OAR proximity, 11 patients were treated primarily for respiratory motion management, and one patient was treated with MRgRT due to a variable pleural effusion. All 11 patients meeting HILUS grouping definition were treated with MRgRT primarily due to OAR proximity. **Figure 1** shows an example for HILUS grouping B due to proximity to the left upper lobe bronchus.

**Table 1:** Cohort characteristics.

Age (median and IQR)	71 (58–80)
**ECOG** 0 1	8 (30%) 19 (70%)
**Sex** Male Female	12 (44%) 15 (56%)
**Histology** NSCLC Other	16 (59%) 11 (41%)
**Stage** I, II, III IV	11 (41%) 16 (56%)
**Concurrent Systemic Therapy** None Immunotherapy Chemotherapy Biologic	18 (67%) 6 (22%) 1 (4%) 2 (7%)
**Expanded HILUS Classification** A B C D NA	6 (22%) 1 (4%) 2 (7%) 2 (7%) 16 (60%)
**Site** Right lung Left lung Mediastinum	15 (55%) 8 (30%) 4 (15%)
**Reason for MRgRT Treatment** Adjacent OAR Motion Management Other	15 (55.6%) 11 (40.7%) 1 (3.7%)

The median dose/fractionation was 50 Gy/5 fractions and 30% (n=51) of fractions delivered were adapted (**Table 2**). Of the 51 adapted fractions, 19 (37.3%) were adapted due to OAR dose, 9 (17.6%) were adapted due to PTV coverage, and the remaining 23 fractions (45.1%) were adapted due to both PTV coverage and OAR dose considerations. For patients treated with MRgRT due to OAR proximity (n=15), 80% had at least 1 adapted fraction during their course of radiotherapy. There was no plan adaptation for patients treated due to motion management (n=11). Nine patients had adapted plans in either their first or final fraction, resulting in different treatment plan times thus impacting duty cycle. For these 9 patients, the average change in treatment plan time from first to final fraction was 0.29 minutes, with only 3 patients plan time changing by more than one minute.

**Table 2:** Treatment characteristics (median and IQR).

**Dose per fraction (Gy)**	10 (7–10)
**Fraction number**	5 (5–5)
**Total dose (Gy)**	50 (50–50)
**Hotspot in GTV (%)**	122 (117–129)
**GTV (cc)**	10.3 (5.43 – 22.5)
**GTV motion (mm)**	9 (4–13)
**PTV expansion (mm)**	5 (5–5)
**PTV volume (cc)**	32.1 (14.0–48.3)
**Number of Adapted Fractions**	0 (0–3)
**Plan Time Fraction 1 (minutes)**	10.0 (8.7–12.5)
**Delivery Time Fraction 1 (minutes)**	17.3 (15.1–28.8)
**Duty Cycle Fraction 1 (%)**	55 (40.3–68.3)
**Plan Time Final Fraction (minutes)**	10.0 (8.7 – 12.5)
**Delivery Time Final Fraction (minutes)**	16.0 (13.5–21.5)
**Duty Cycle Final Fraction (%)**	64.1 (54.7 – 73.7)

**Figure 2:**
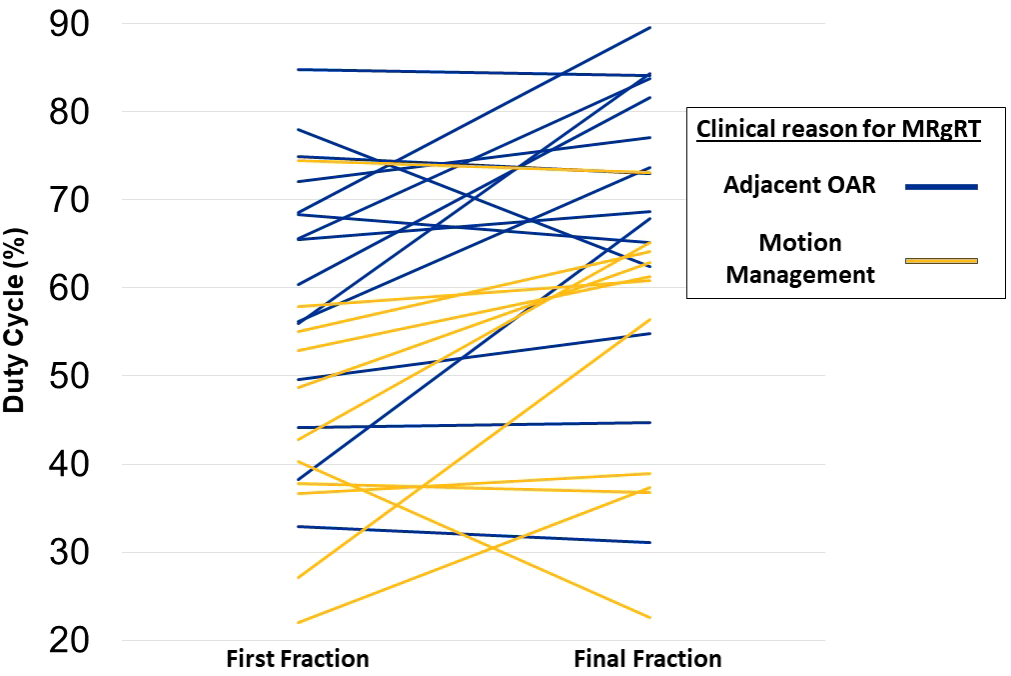
Change in duty cycle between first and final fraction. Each line represents a single patient and color denotes if a patient was treated primarily due to motion management or adjacent organ at risk.

Mean GTV motion was significantly higher for patients treated due to motion management compared to OAR (16.1mm vs. 6.5mm, p=0.011). Mean duty cycle for fraction 1 was 54.2% compared to 62.1% for the final fraction (p=0.0035). In the subset of 18 patients with identical treatment plan times in fraction 1 and final fraction (i.e. fraction 1 and final fraction were not adapted), mean duty cycle for fraction 1 was 52.6% compared to 60.9% for the final fraction (p=0.0075). Duty cycle was higher for patients treated due to OAR compared to patients treated for motion management in fraction 1 (61% vs. 45.0%, p=0.0124) and in the final fraction (69.5% vs. 52.7%, P=0.0146; **Figure 2**). The mean duty cycle for fraction 1 was 65.3% for patients treated before installation of the visual feedback display compared to 49.6% for patients treated after installation (p=0.0185). The mean duty cycle for final fraction was 68.0% for patients before visual feedback display installation compared to 59.6% for patients without (p=0.2657).

## 4 Discussion

During our institution’s MRgRT program, we delivered thoracic MRgRT to 27 patients over 169 fractions for a variety of intrathoracic tumors. We found that on average, treatment duty cycle improved by an average of 8% between fraction 1 and the final fraction. Since the time for beam delivery, multileaf collimator (MLC) motion, and gantry rotation are mostly constant, the improvement likely correlates with patient breath hold performance between first and final fractions.

The duty cycle plays a critical role in the overall efficacy of MRgRT respiratory gated treatments. Treatment times are significantly longer for patients undergoing real time tracking and breath-hold gating on MR-Linac when compared to conventional linac treatments. This leads to a higher probability for changes in patient positioning during treatment that may not be reflected in a 2D cine image, changes in patients breathing acumen due to duration and fatigue, and possibility of respiratory baseline shift during treatment, which may change target motion trajectory. The factors contributing to duty cycle are complex and include medical factors, (baseline cardiopulmonary function impacting breath hold duration), psychological factors (patient understanding and education), and technical factors (tumor, MLC, and gantry motion). Thus, patient selection and continual patient education is key to maximize the treatment duty cycle.

Our work adds to the literature describing the feasibility of using MRgRT for thoracic tumors and adds hypothesis-generating results about the improvement in duty cycle between first and final fraction. This likely reflects improved patient familiarity with the treatment and breath hold technique needed on subsequent fractions to accurately get the tumor into the target region. The therapist team debriefed the patient after each fraction to answer questions and offer suggestions to improve treatment efficiency. Additionally, a physics consult was offered to MRgRT patients prior to simulation, in which breath hold gating was discussed with visual aids. Further analysis is required to validate the impact of physics consultation on duty cycle efficiency.

Respiratory motion is complex and there is variability in the extent of craniocaudal and anteroposterior displacement between fractions (3). In our clinical observations, we noted that several patients have a striking change in target location as the patient relaxed into their breath hold, which necessitates them breathing their tumors past the region of interest prior to breath hold initiation (**Appendix Video 1**). This observation is not well-described in the current literature. This may confuse patients using visual feedback displays who are attempting to move their tumors into the region of interest. This observation is supported by the decreased duty cycle for patients treated after to installation of the visual feedback display. However, this association needs to be analyzed with a broader scope, as some patients chose not to fully utilize the visual feedback display and majority of patients in our cohort treated after visual feedback display installation were being treated due to complex motion management as opposed to OAR proximity. In the depicted patient in the **Appendix Video 1**, duty cycle was improved from first fraction (52.8%) to final fraction (61.3%).

There have been several published series utilizing MRgRT for thoracic tumors. One group evaluated “treatment process time efficiency” across multiple disease sites, calculated in a similar fashion to duty cycle in the present study, though the time for gantry rotation and MLC motion were not included. Two-hundred and sixty-eight fractions were treated with deep inspiration breath hold with a 42.4%treatment process time efficiency (8). Adjusting our calculations to exclude gantry rotation and MLC motion yields a similar value, with a 41.3% average duty cycle for all fractions, 37.0% average duty cycle for fraction 1 and 45.6% final fraction average duty cycle. Another study examined 15 patients treated with 87 fractions to lung, adrenal, and pancreatic tumors, and evaluated “duty cycle efficiency”, defined as the total number of “beam-on” frames divided by the total number of MR cine frames acquired during treatment delivery (9). The mean duty cycle efficiency for lung tumors was 68.7%, which was significantly higher than our calculated duty cycle. We believe this is primarily due to differences in threshold region of interest (ROI), which is the maximum percentage of the target that can be out of the tracking volume without stopping radiation delivery. The threshold-ROI in the study varied from 10% - 20% for the patients with lung tumors, whereas all of the patients treated within our study utilized a threshold-ROI<8%, with majority utilizing 5%. The tighter threshold increases the difficulty of precisely breathing the tumor into the ROI, likely resulting in the decreased mean duty cycle comparatively seen in our study. Another study of 14 patients with 15 lung tumors treated with MRgRT reported a 53% median treatment duty cycle, though the duty cycle calculation method was not reported (10). Other studies in the thoracic MRgRT space evaluated general feasibility and potential benefits of adaptation (11), peripheral tumor motion in breath-hold vs. free breathing (12), safety and feasibility of single fraction SBRT with MRgRT (13), and MRI-based lung tumor motion. The aforementioned studies focus on evaluating overall tumor size and its effect on motion, validating tumor motion models, reproducibility, and surrogate and fiducial based tracking (14–20).

Several studies have been published on using MRgRT for higher risk treatment of intrathoracic tumors (21, 22). One study evaluated 50 patients receiving MRgRT for high risk tumors defined as centrally located, previous thoracic radiotherapy, or interstitial lung disease (21). Ablative radiation (BED ≥ 100) was delivered to over 90% of tumors and 12 month local control was 95.6%, with only 8% grade 3 toxicity and no grade 4 or 5 toxicities. Another study of 47 patients with central (n=21) or ultra-central (n=26) tumors were treated with MRgRT to a median dose of 60 Gy in 8 fractions. Reported 1 year local control was 87% with only 2 late grade 3 toxicities (4.3%) and no grade 4 or 5 toxicities in this high risk patient population (22). The therapeutic index described in these studies favorably compares to the recent Nordic HILUS study of CT-based radiation for central lung tumors, where 30% of patients experienced grade 3–5 toxicity with a treatment related death rate of 15% (4). Notably, the HILUS trial did not utilize breath-hold MRgRT and thus treatment volumes were inherently larger and without real-time tumor tracking. Additionally, lobar bronchi and great vessels were not contoured as avoidance OARs, walls of luminal OARs were not included, and hotspots in radiotherapy plans were 150%, which may have contributed to excessive radiation dose to critical OARs and observed toxicity rates (23). Future phase II studies should be conducted to prospectively validate MRgRT for high risk intrathoracic tumors.

Some limitations of our work should be discussed. First, we report 30% of fractions were adapted, but the decision to adapt was at the treating physician’s discretion. This is in contrast to some series where all plans were adapted. Generally, plans may not have been adapted despite small differences in coverage or OAR dose that may have minimally exceeded pre-specified planning goals if not deemed clinically significant by the treating physician. Additionally, it is unclear how the addition of visual aid may have truly influenced duty cycle, requiring a more detailed data analysis outside the scope of this paper. Lastly, this report covers only the technical treatment data of our patient cohort; clinical outcomes such as tumor control and toxicity have not been collected.

## 5 Conclusions

MRgRT was utilized to treat 27 patients over 169 fractions for various intrathoracic tumors. There was a significant improvement in treatment duty cycle between the first and final fraction possibly due to continued patient education and familiarity with treatment. MRgRT is a feasible modality to treat intrathoracic tumors though future work should be conducted to further optimize duty cycle and thus improve treatment efficiency.

## Data availability statement

The raw data supporting the conclusions of this article will be made available by the authors, without undue reservation.

## Ethics statement

The studies involving humans were approved by Institutional Review Board (IRB). The studies were conducted in accordance with the local legislation and institutional requirements. Written informed consent for participation was not required from the participants or the participants’ legal guardians/next of kin in accordance with the national legislation and institutional requirements.

## Author contributions

JM: Conceptualization, Data curation, Formal analysis, Investigation, Methodology, Project administration, Resources, Software, Supervision, Validation, Visualization, Writing – original draft, Writing – review & editing. NP: Conceptualization, Data curation, Formal analysis, Investigation, Methodology, Software, Writing – original draft, Writing – review & editing, Supervision. AS: Data curation, Formal analysis, Investigation, Resources, Validation, Writing – review & editing. MY: Data curation, Formal analysis, Writing – review & editing. SM: Data curation, Formal analysis, Writing – review & editing. MF: Data curation, Formal analysis, Writing – review & editing. KS: Data curation, Formal analysis, Writing – review & editing. AD: Data curation, Formal analysis, Writing – review & editing. JK: Data curation, Formal analysis, Writing – review & editing. AB: Data curation, Formal analysis, Writing – review & editing. LT: Data curation, Formal Analysis, Writing – review & editing. MM: Conceptualization, Data curation, Formal analysis, Methodology, Project administration, Writing – review & editing.

## Supplementary material

Appendix Video 1 74-year-old former heavy smoker with a cT1bN0 NSCLC of the right lower lung. He received 50Gy in 5 fractions utilizing MRgRT due to motion management as GTV respiratory motion was 1.3cm. When he relaxes into his breath hold, the tumor drifts superiorly and sometimes out of the gating boundary. He was educated to breathe his tumor past the ROI for subsequent treatments. His duty cycle improved from first fraction (52.8%) to final fraction (61.3%).
