# Supplementary figures and images for: Single institution experience of MRI-guided radiotherapy for thoracic tumors and clinical characteristics impacting treatment duty cycle
Source: Front Oncol. 2024 Jun 11;14:1401703. doi: 10.3389/fonc.2024.1401703 (PMC11196615; doi:10.3389/fonc.2024.1401703)

## Slide 1
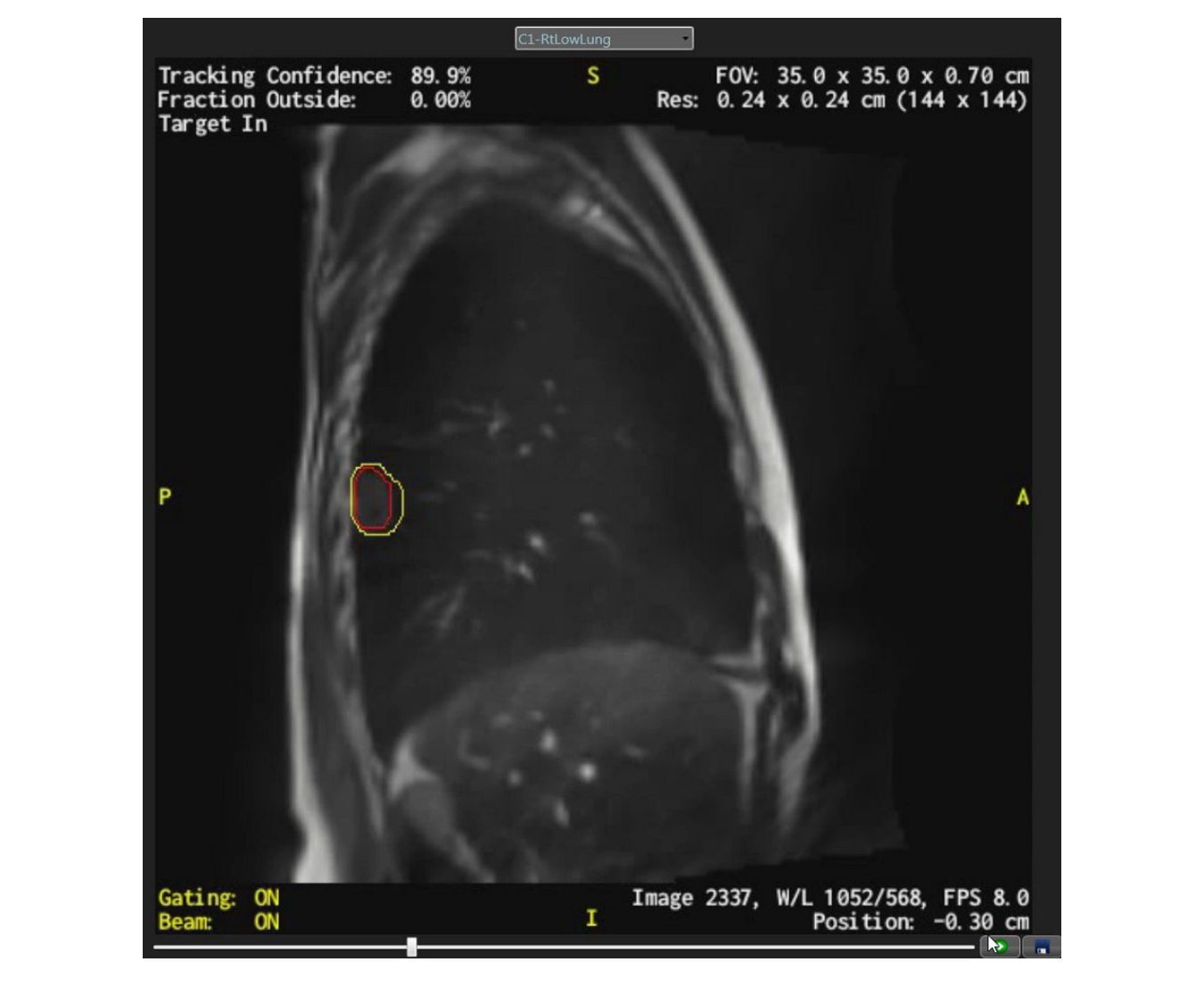

Supplement: Appendix Video 1 — 74-year-old former heavy smoker with a cT1bN0 NSCLC of the right lower lung. He received 50Gy in 5 fractions utilizing MRgRT due to motion management as GTV respiratory motion was 1.3cm. When he relaxes into his breath hold, the tumor drifts superiorly and sometimes out of the gating boundary. He was educated to breathe his tumor past the ROI for subsequent treatments. His duty cycle improved from first fraction (52.8%) to final fraction (61.3%). [file Presentation_1.pptx]
